# Role of Ezrin Phosphorylation in HIV-1 Replication

**DOI:** 10.3389/fmicb.2018.01912

**Published:** 2018-08-27

**Authors:** Haruka Kamiyama, Mai Izumida, Yuria Umemura, Hideki Hayashi, Toshifumi Matsuyama, Yoshinao Kubo

**Affiliations:** ^1^Department of AIDS Research, Institute of Tropical Medicine, Nagasaki University, Nagasaki, Japan; ^2^Department of Molecular Microbiology and Immunology, Graduate School of Biomedical Sciences, Nagasaki University, Nagasaki, Japan; ^3^Department of Clinical Medicine, Institute of Tropical Medicine, Nagasaki University, Nagasaki, Japan; ^4^Medical University Research Administrator (MEDURA), Nagasaki University School of Medicine, Nagasaki, Japan; ^5^Department of Cancer Stem Cell Biology, Institute of Biomedical Sciences, Nagasaki University, Nagasaki, Japan; ^6^Program for Nurturing Global Leaders in Tropical and Emerging Communicable Diseases, Graduate School of Biomedical Sciences, Nagasaki University, Nagasaki, Japan

**Keywords:** HIV-1, ezrin, viral entry, viral assembly, viral budding

## Abstract

Host-cell expression of the ezrin protein is required for CXCR4 (X4)-tropic HIV-1 infection. Ezrin function is regulated by phosphorylation at threonine-567. This study investigates the role of ezrin phosphorylation in HIV-1 infection and virion release. We analyzed the effects of ezrin mutations involving substitution of threonine-567 by alanine (EZ-TA), a constitutively inactive mutant, or by aspartic acid (EZ-TD), which mimics phosphorylated threonine. We also investigated the effects of ezrin silencing on HIV-1 virion release using a specific siRNA. We observed that X4-tropic HIV-1 vector infection was inhibited by expression of the EZ-TA mutant but increased by expression of the EZ-TD mutant, suggesting that ezrin phosphorylation in target cells is required for efficient HIV-1 entry. Expression of a dominant-negative mutant of ezrin (EZ-N) and ezrin silencing in HIV-1 vector-producing cells significantly reduced the infectivity of released virions without affecting virion production. This result indicates that endogenous ezrin expression is required for virion infectivity. The EZ-TD but not the EZ-TA inhibited virion release from HIV-1 vector-producing cells. Taken together, these findings suggest that ezrin phosphorylation in target cells is required for efficient HIV-1 entry but inhibits virion release from HIV-1 vector-producing cells.

## Introduction

Human immunodeficiency virus type 1 (HIV-1) is the etiological agent of AIDS. In addition, the replication-defective HIV-1 vector is frequently used as a gene transfer tool in many biological fields of study. The HIV-1 vector is generally constructed by transfection of 293T or COS7 fibroblast cell lines that express the tumor antigen of simian virus 40 and have high transfection efficiency. Understanding the molecular mechanism of HIV-1 replication in these cell lines would provide novel approaches to obtaining high-efficiency HIV-1 vectors and developing new therapeutic strategies against AIDS.

Many cytoskeleton-associated cellular factors are involved in the entry of HIV-1 into host cells (Jiménez-Baranda et al., 2007; Naghavi et al., 2007; Barrero-Villar et al., 2009; Capalbo et al., 2011; Gordón-Alonso et al., 2013). We have reported that an ezrin dominant-negative mutant and siRNA-mediated knockdown of ezrin expression in target cells inhibit infection by the envelope protein (Env) of CXCR4 (X4)-tropic HIV-1. This observation demonstrates that ezrin is required for X4-tropic HIV-1 infection (Kubo et al., 2008). Moesin, another member of the ezrin-radixin-moesin (ERM) family, is also necessary for HIV-1 entry (Barrero-Villar et al., 2009). Furthermore, ezrin protein is efficiently incorporated into HIV-1 particles, suggesting a role of ezrin in HIV-1 virion production (Linde et al., 2013).

Ezrin protein function is primarily regulated by phosphorylation of the threonine residue at position 567 (Tsukita and Yonemura, 1997; Matsui et al., 1998). The N- and C-terminal domains of unphosphorylated ezrin are intramolecularly and/or intermolecularly bound to each other. After ezrin phosphorylation, the interaction between these domains is abolished, allowing the binding of the N- and C-terminal termini to their membrane protein targets and the actin cytoskeleton, respectively. Because an N-terminal domain peptide of ezrin (EZ-N) binds to the membrane proteins but does not interact with the actin cytoskeleton, it acts as a dominant negative mutant of phosphorylated ezrin. Although ezrin is involved in HIV-1 entry and virion production, role of the ezrin phosphorylation in HIV-1 replication is unknown.

This study investigates the impact of ezrin phosphorylation in X4-tropic HIV-1 infection and virion release using ezrin mutants. Mutants containing amino acid substitutions of the threonine-567 residue for alanine (EZ-TA) and aspartic acid (EZ-TD) were constructed. EZ-TA functions as a constitutively inactive mutant, because it cannot be phosphorylated. EZ-TD functions as a constitutively active mutant, because the aspartic acid residue mimics phosphorylated threonine. We investigated the effect of these mutants on HIV-1 virion production.

## Materials and Methods

### Expression Plasmids

The HIV-1 gag, pol, tat, and rev expression plasmid (R8.91) was kindly provided by Dr. D. Trono (Naldini et al., 1996). The LacZ-encoding HIV-1 vector genome expression plasmid and VSV-G expression plasmid were obtained from Dr. L.J. Chang through the AIDS Research and Reference Reagent Program, NIAID, NIH, United States (Chang et al., 1999; Iwakuma et al., 1999). The X4-tropic HIV-1 HXB2 Env expression plasmid was kindly provided by Dr. Y. Yokomaku (Yokomaku et al., 2004). The VSV-G epitope-tagged ezrin expression plasmid was obtained from Dr. M. Arpin (Algrain et al., 1993). The CD4-expressing lentiviral vector (Invitrogen) was constructed in our laboratory. MLV vectors (Kitamura, 1998) encoding VSV-G epitope-tagged EZ-Wt, -N, -TA, and -TD were constructed in this study. The infectious molecular clone HIV-1 NL4-3 was kindly provided by Dr. A. Adachi (Nomaguchi et al., 2014).

### Cells

COS7, HeLa, and 293T cells were cultured with Dulbecco’s modified Eagle’s medium (D-MEM) (Wako) supplemented with 8% fetal bovine serum at 37°C in 5% CO_2_. CD4-expressing HeLa cells (HeLa/CD4) were constructed as follows. COS7 cells were transfected with the R8.91 (Naldini et al., 1996), VSV-G (Iwakuma et al., 1999), and CD4-encoding lentiviral vector expression plasmids. Culture supernatants of the transfected cells were inoculated into HeLa cells. Because the lentiviral vector encodes the blastidine resistance gene, the inoculated cells were selected with blastidine (Invitrogen). The blastidine-resistant cell pool was used in this study. HeLa/CD4 cells were further inoculated with a MLV vector (Kubo et al., 2003) encoding EZ-Wt, -N, -TA, or -TD. Because the MLV vector additionally encodes the puromycin resistance gene, the inoculated cells were selected with puromycin; puromycin-resistant cell pools were used in this study.

### HIV-1 Vector

COS7 cells were transfected with the R8.91 (Naldini et al., 1996), lacZ-encoding HIV-1 vector genome (Chang et al., 1999), and HXB2 Env expression (Yokomaku et al., 2004) plasmids together with one of the ezrin expression plasmids using the Fugene HD transfection reagent (Promega). The transfected cells were washed with fresh medium to remove the transfection complex 24 h after transfection and cultured for additional 24 h. Culture supernatants of the transfected cells were inoculated onto target cells. The cells were stained with 5-bromo-4-chloro-3-indolyl-b-D-galactopyranoside (X-Gal) (Wako) 2 days after inoculation. Blue cells were counted in 8 randomly selected microscopic fields per a 3-cm culture dish. Total of the blue cell numbers were calculated as transduction titers. Total blue cell numbers in control cells were always set to 1. Relative values to the blue cell numbers in control cells were calculated. The experiment was repeated three times. Usually 100∼300 blue cells were detected in control cells (Kamiyama et al., 2009; Yoshii et al., 2011).

### Western Immunoblotting

Culture supernatants of the vector-producing cells were filtered through a 0.45-μm membrane (Millipore), and centrifuged at 16,114 × *g* through 20% sucrose for 5 h to collect virion pellets. Cell lysates and virion pellets were subjected to SDS polyacrylamide gel electrophoresis with or without Phos-tag reagent (Kinoshita et al., 2006), and proteins were transferred onto a PVDF membrane. Membranes treated with rabbit anti-HIV-1 p24 (BioAcademia or ZeptoMetrix), sheep anti-HIV-1 gp120 (provided by Dr. T. Murakami), or rabbit anti-ezrin antibody (Santa Cruz Biotechnology) then were treated with HRP-conjugated protein G (BioRad) to detect the proteins. Membranes treated with mouse anti-VSV-G epitope (Sigma-Aldrich) and mouse anti-actin antibodies (Santa Cruz Biotechnology) were treated with HRP-conjugated anti-mouse IgG (BioRad) as the secondary antibody. Antigen proteins were visualized using the Clarity Western ECL substrate (BioRad).

### Site-Directed Mutagenesis

Site-directed mutagenesis was performed using the standard PCR-mediated protocol (TaKaRa). The primers were synthesized by Fasmac Co., The nucleotide sequences of the resulting plasmids were confirmed (Applied Biosystems).

### Virus-Cell Membrane Fusion Activity

Virus-cell membrane fusion activity was measured as previously reported (Cavrois et al., 2002). COS7 cells were transfected with the HIV-1 vector construction plasmids and a plasmid encoding the BlaM-Vpr fusion protein together with pcDNA3.1, EZ-Wt, EZ-N, or siEZ. HeLa/CD4 cells were inoculated with culture supernatants from the transfected cells and stained with CCF2 (Invitrogen). Intact CCF2 releases fluorescence at 450 nm. When CCF2 is cleaved by BlaM-Vpr, the product releases fluorescence at 405 nm. Fluorescence intensities at 450 and 405 nm of the cells were measured using a microplate fluorometer (Perkin Elmer), and ratios of fluorescence intensities at 405 nm to those at 450 nm were calculated. When HIV-1 vector particles containing BlaM-Vpr enter into target cells, the fluorescence ratios are increased.

### Cellular Localization of HIV-1 Gag and Ezrin Proteins

Transfected cells were permeated by methanol and stained with rabbit anti-HIV-1 p24 and mouse anti-VSV-G epitope antibodies. The cells then were treated with FITC-conjugated anti-rabbit IgG and Cy3-conjugated anti-mouse IgG antibodies. The cells were observed under a confocal fluorescent microscopy (Zeiss).

### HIV-1 Replication

293T cells were transfected with the infectious molecular clone of HIV-1 NL4-3. Target cells were inoculated with culture supernatants (10 μl) of the transfected cells. Inoculated cells were changed to fresh medium 1 day after inoculation. Culture supernatant concentrations of HIV-1 Gag p24 were measured by ELISA (ZeptoMetrix) 3 days after the inoculation.

### Statistical Analysis

Differences between two groups of data were determined using Student’s *t*-test. Statistical significance was set at *p* < 0.05 for all tests.

## Results

### Ezrin Phosphorylation in Target Cells Is Required for Efficient HIV-1 Infection

**FIGURE 1:**
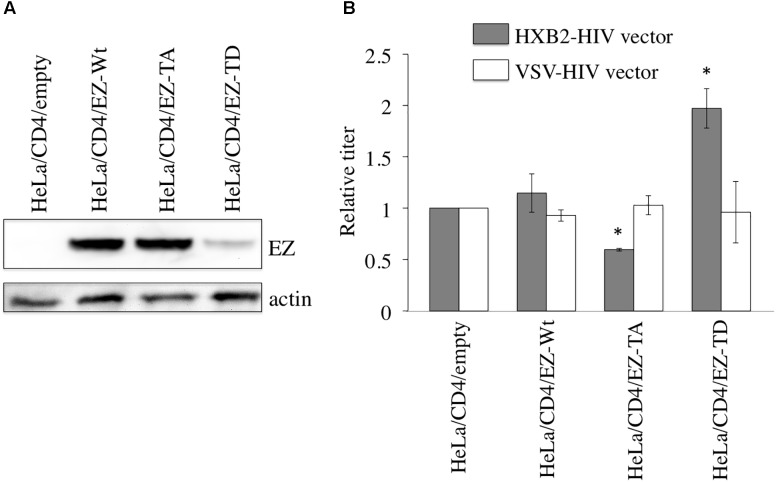
Ezrin phosphorylation in target cells is required for efficient X4-tropic HIV-1 infection. **(A)** Expression levels of C-terminally VSV-G epitope-tagged EZ-Wt, EZ-TA, and EZ-TD proteins in HeLa/CD4 cells were analyzed by western immunoblotting using the anti-VSV-G epitope antibody (upper panel). Actin protein served as a control (lower panel). **(B)** Transduction titers of the X4-tropic Env- and VSV-G-pseudotyped HIV-1 vectors were measured for the empty vector-, EZ-Wt-, EZ-TA-, and EZ-TD-expressing HeLa/CD4 cells. Transduction titers of the empty vector-expressing cells were always set to 1, and relative values of transduction titers in other cells are indicated. Error bars show standard deviations, and asterisks indicate statistically significant differences compared to the empty vector-expression cells.

To assess whether ezrin phosphorylation in target cells is required for HIV-1 infection, murine leukemia virus (MLV) vector encoding C-terminally VSV-G epitope-tagged ezrin wild type (EZ-Wt) (Algrain et al., 1993), EZ-TA, and EZ-TD were constructed. The number of puromycin-resistant cell colonies was lower in those inoculated with the EZ-TD-expressing MLV vector than with the EZ-Wt- or EZ-TA-encoding vector. Western blot analysis revealed that the amount of EZ-TD protein was less than that of EZ-Wt or EZ-TA (**Figure 1A**), suggesting that EZ-TD might inhibit cell proliferation or have cytopathic activity in HeLa cells.

HIV-1 vectors containing X4-tropic HXB2 Env or VSV-G protein were inoculated onto these puromycin-resistant cell pools. The transduction titers of the X4-tropic Env-containing HIV-1 vector in EZ-TA-expressing cells were moderately lower than those in empty vector- or EZ-Wt-expressing cells (**Figure 1B**). In contrast, the transduction titers of the X4-tropic Env-containing vector in EZ-TD-expressing cells were higher than those of control cells. The transduction titers of VSV-G-pseudotyped HIV-1 vector were comparable in all of these cell pools. These results suggest that ezrin phosphorylation in target cells is required for the efficient X4-tropic Env-mediated infection.

### Ezrin Dominant Negative Mutant Incorporated Into HIV-1 Virions Inhibits Infection

**FIGURE 2:**
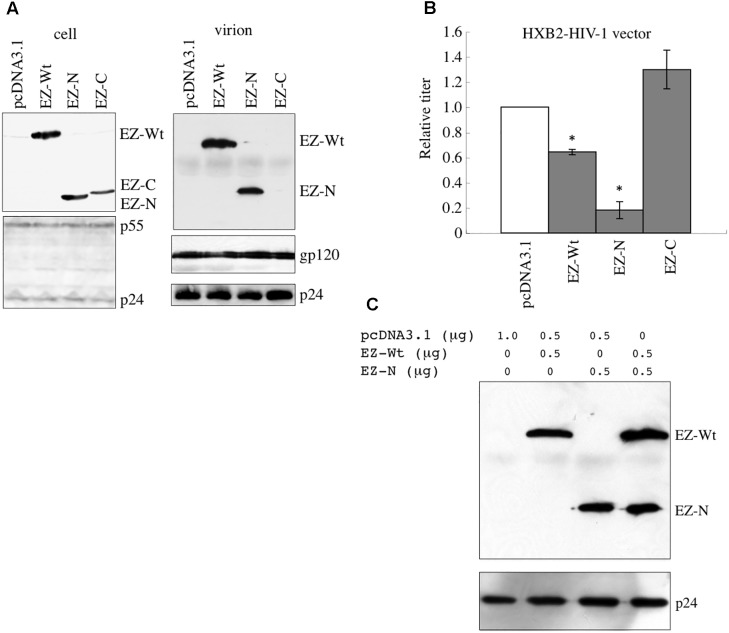
Incorporation of EZ-N into HIV-1 vector particles reduces their infectivity. **(A)** COS7 cells were transfected with the HIV-1 vector construction plasmids together with the pcDNA3.1, EZ-Wt, EZ-N, or EZ-C expression plasmid. Cell lysates (left panel) and virion-containing pellets of culture supernatants from the transfected cells (right panel) were analyzed by western immunoblotting using the anti-VSV-G epitope (upper panel) or anti-HIV-1 p24 (lower panel) antibody. (**B**) Culture supernatants from the transfected cells were inoculated into HeLa/CD4 cells, and transduction titers were measured. Transduction titers of pcDNA3.1-transfected cells were always set to 1, and relative values of transduction titers of other cells are indicated. Error bars show standard deviations, and asterisks indicate statistically significant differences compared to the pcDNA3.1-transfected cells. (**C**) COS7 cells were transfected with the HIV-1 vector construction plasmids together with EZ-Wt and/or EZ-N. Virion pellets from the transfected cells were analyzed by western immunoblotting using the anti-VSV-G epitope or anti-p24 antibody.

It has been reported that ezrin protein is incorporated into HIV-1 virion (Ott et al., 1996). To analyze roles of ezrin in the HIV-1 virion production and infectivity of released particles, COS7 cells were transfected by the X4-tropic HIV-1 vector construction plasmids together with the plasmid encoding the EZ-Wt, N-terminal region (EZ-N), or C-terminal region (EZ-C). Western blot analysis of cells transfected with X4-tropic HIV-1 vector together with EZ-Wt, EZ-N, or EZ-C revealed that the amount of HIV-1 Gag protein is similar for all transfected constructs (**Figure 2A**). This result indicates that EZ-Wt, -N, and -C expression do not affect HIV-1 virion release.

Western blot analysis of virion pellets using anti-VSV-G epitope antibody revealed the presence of EZ-Wt and EZ-N but not EZ-C protein (**Figure 2A**, right panel), suggesting that the N-terminus is responsible for ezrin incorporation into viral particles.

While the transduction titers of culture supernatants from EZ-N-transfected cells were significantly lower than those of pcDNA3.1-transfected cells (**Figure 2B**), the levels of HIV-1 p24 and gp120 in virion pellets did not differ (**Figure 2A**). The transduction titers of culture supernatants from EZ-Wt–transfected cells were slightly lower than those of control cells. EZ-C was not incorporated into virions, and its expression did not change the transduction titers. These results indicate that incorporation of the dominant negative EZ-N protein into virions decreases the infectivity of released HIV-1 particles.

The effect of EZ-N expression on the incorporation of wild-type ezrin protein into HIV-1 vector was examined. Cells transfected with HIV-1 vector together with both EZ-Wt and EZ-N incorporated the same amount of EZ-Wt protein in virion pellets as those transfected with EZ-Wt alone (**Figure 2C**), indicating that EZ-N has no effect on EZ-Wt incorporation into HIV-1 particles.

### Ezrin Silencing Decreases the Infectivity of Released HIV-1 Particles

**FIGURE 3:**
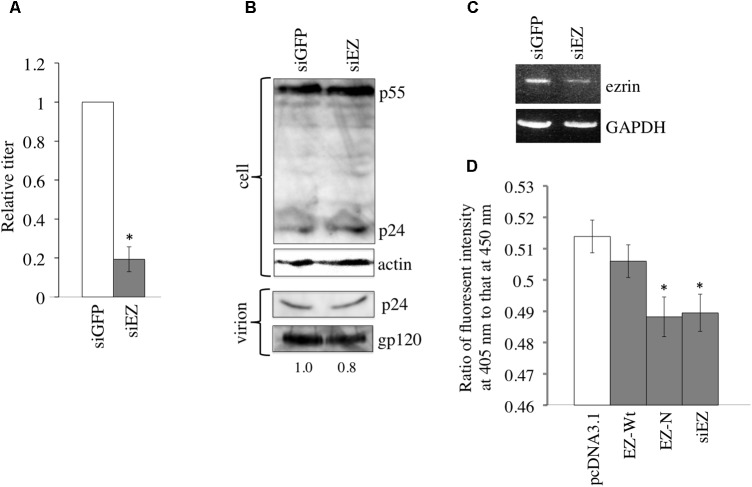
Ezrin is required in HIV-1 vector-producing cells for infectivity of released vector particles. **(A)** 293T cells were transfected with the HIV-1 vector construct plasmids together with siGFP or siEZ, and culture supernatants from the transfected cells were inoculated onto HeLa/CD4 cells. Transduction titers of siGFP-transfected cells were always set to 1, and relative values of transduction titers are indicated. Error bar shows standard deviations, and an asterisk indicates a statistically significant difference compared to siGFP-transfected cells. **(B)** Cell lysates (upper panel) and virion pellets of culture supernatants from transfected cells (lower panel) were analyzed by western immunoblotting using the anti-HIV-1 p24 or anti-actin antibody. Intensities of gp120 protein bands were measured, and relative values to gp120 intensity in siGFP-transfected cells were indicated. **(C)** Semi-quantitative RT-PCR was performed on transfected cells to measure ezrin and GAPDH mRNA levels. **(D)** Virus-cell fusion activity of indicated HIV-1 vector particles was measured. Ratios of fluorescence intensities at 405 nm to those at 450 nm are indicated. Error bars show standard deviations, and asterisks indicate statistically significant differences compared to the values of pcDNA3.1 HIV-1 vector.

To assess whether endogenous ezrin in HIV-1 vector-producing cells is required for HIV-1 virion production and/or infectivity of released HIV-1 virions, 293T cells were transfected with X4-tropic HIV-1 vector together with an siRNA against GFP (siGFP) or ezrin (siEZ). The transduction titers of culture supernatants from siEZ-transfected cells were significantly lower than those from siGFP-transfected cells (**Figure 3A**), consistent with a previous report (Roy et al., 2014). The amount of HIV-1 Gag protein in cell lysates and virion pellets was not altered by siEZ (**Figure 3B**). The amount of HIV-1 gp120 in virion pellets was not significantly changed. The ezrin knockdown by the siEZ was confirmed by RT-PCR but not western blotting, because anti-ezrin antibodies generally detect radixin, another member of ERM family as well as ezrin and their molecular sizes are same (Algrain et al., 1993; Kubo et al., 2008). siEZ transfection decreased the amount of ezrin mRNA (**Figure 3C**). These results show that ezrin is dispensable for HIV-1 virion production but required for the infectivity of released HIV-1 virions.

To assess the effect of EZ-N and ezrin silencing on X4-tropic Env-mediated entry, we investigated the fusion of the virus-cell membranes. EZ-N and siEZ but not EZ-Wt attenuated virus-cell fusion (**Figure 3D**), indicating that the decreased infectivity of HIV-1 particles from EZ-N- and siEZ-expressing cells is related to the suppression of virus-cell membrane fusion.

### Phosphorylated Ezrin Suppresses HIV-1 Virion Release

**FIGURE 4:**
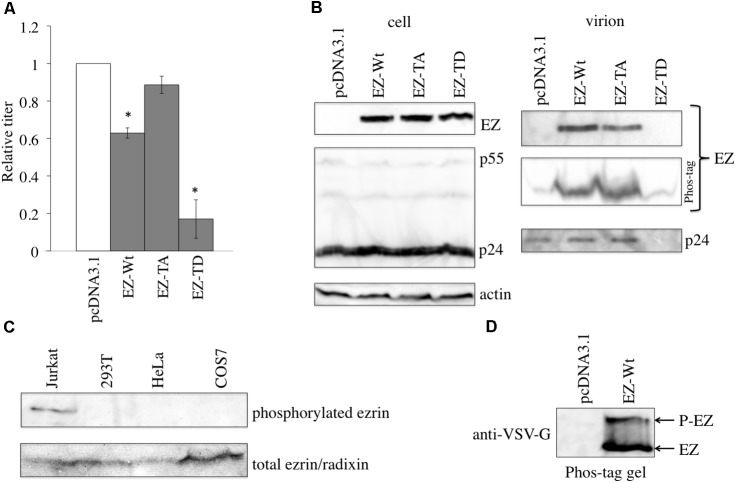
EZ-TD inhibits HIV-1 vector release. **(A)** COS7 cells were transfected with the HIV-1 vector construction plasmids together with pcDNA3.1, EZ-Wt, EZ-TA, or EZ-TD expression plasmid, and culture supernatants from the transfected cells were inoculated onto HeLa/CD4 cells. Transduction titers of pcDNA3.1-transfected cells were always set to 1, and relative values of transduction titers of other transfected cells are indicated. Error bars show standard deviations, and asterisks indicate statistically significant differences compared to pcDNA3.1-transfected cells. **(B)** Cell lysates (left panels) and virion pellets of culture supernatants from the transfected cells (right panels) were analyzed by western immunoblotting using the anti-VSV-G epitope, anti-HIV-1 p24, or anti-actin antibody. Virion pellets were also separated on Phos-tag reagent-containing gels. **(C)** Cell lysates from Jurkat, 293T, HeLa, and COS7 cells were analyzed by western immunoblotting using the anti–phosphorylated-ezrin (upper panel) or anti-ezrin (lower panel) antibody. **(D)** Cell lysate from Jurkat cells transfected by the EZ-TD expression plasmid were applied to Phos-Tag reagent-containing gel, and then analyzed by western blotting using the anti-VSV-G epitope antibody.

To analyze the effect of ezrin phosphorylation on virion release, COS7 cells were transfected with X4-tropic HIV-1 vector together with pcDNA3.1, EZ-Wt, EZ-TA, or EZ-TD. HIV-1 vector infectivity was not affected by EZ-TA expression and only slightly decreased by EZ-Wt expression (**Figure 4A**). In contrast, EZ-TD expression dramatically decreased transduction titers. Although the amount of HIV-1 Gag protein expressed in the transfected cells was not affected by EZ-Wt, EZ-TA, or EZ-TD expression, no p24 protein was observed in supernatants of EZ-TD–expressing cells (**Figure 4B**, right panel). The observed suppression of virion release by EZ-TD suggests that phosphorylated ezrin inhibits such release.

The phosphorylation status of endogenous ezrin was investigated by western blot analysis. Phosphorylated ezrin was detected in Jurkat T cells, but not in 293T, HeLa, or COS7 cells (**Figure 4C**), as already reported (Parameswaran and Gupta, 2013). Phosphorylated ezrin protein was not detected in virion pellets from the EZ-Wt-transfected cells. When protein samples are separated in Phos-Tag reagent-containing gel, migration speeds of phosphorylated proteins are moderately reduced than expected (Kinoshita et al., 2006). When cell lysate prepared from Jurkat cells transfected by the EZ-Wt expression plasmid was applied to Phos-Tag reagent-containing gel, a protein with higher molecular size than expected was indeed detected (**Figure 4D**). However, molecular weight of the EZ-Wt protein was similar to that of the EZ-TA protein, with no higher molecular-weight proteins detected in EZ-Wt-expressing cells (**Figure 4B**, right panel). These results indicated that ezrin protein is not phosphorylated in 293T, HeLa, and COS7 cells, and unphosphorylated ezrin is incorporated into HIV-1 virions.

### Phosphorylated Ezrin Inhibits Virion Budding but Not Gag Localization

**FIGURE 5:**
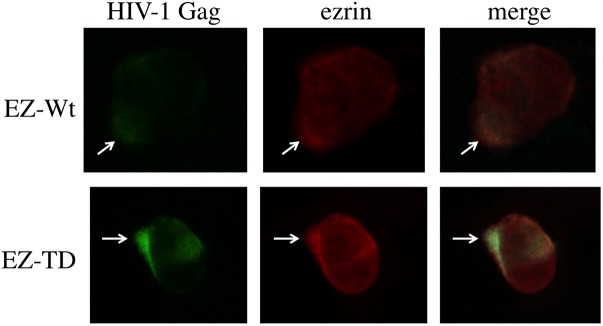
Cellular localization of HIV-1 Gag and ezrin proteins. COS7 cells were transfected with the HIV-1 vector construction plasmids together with the EZ-Wt or EZ-TD expression plasmid, and permeabilized with methanol. The cells were first stained with mouse anti-VSV-G epitope and rabbit anti-HIV-1 p24 antibodies, and then with Cy3-conjugated anti-mouse IgG and FITC-conjugated anti-rabbit IgG antibodies. The cells were observed under a laser confocal microscope. Representative results are shown.

The cell-surface expression of Gag was investigated to determine whether expression of the EZ-TD mutant affects Gag localization. Fluorescence microscopy revealed that EZ-Wt and EZ-TD proteins were concentrated at the aggregated sites of HIV-1 Gag protein (**Figure 5**, arrows) and that the cellular localization of the HIV-1 Gag protein in EZ-TD cells was similar to that of EZ-WT cells (**Supplementary Figure 1**). These results suggest that expression of the EZ-TD mutant did not alter aggregation of the HIV-1 Gag protein.

### EZ-N and siEZ Inhibit HIV-1 Replication

**FIGURE 6:**
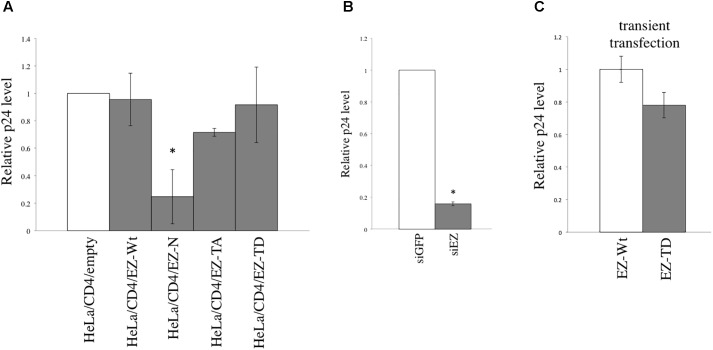
EZ-N and ezrin silencing inhibit HIV-1 replication. **(A)** HIV-1 NL4-3 was inoculated in HeLa/CD4 cells stably expressing EZ-Wt, -N, -TA, -or TD. **(B)** HeLa/CD4 cells were transfected with siGFP or siEZ and then inoculated with HIV-1 NL4-3. Gag p24 concentrations in culture supernatants were measured. p24 levels from the empty vector-transduced cells were always set to 1, and relative values of p24 levels are indicated. Error bars show standard deviations, and asterisks indicate statistically significant differences compared to the values of the empty vector-transduced cells. **(C)** HeLa/CD4 cells were transfected by EZ-Wt or EZ-TD expression plasmid, and were inoculated with replication-competent HIV-1 24 h after the transfection. The p24 levels in culture supernatants were analyzed 3 days after the inoculation.

In the above experiments, replication-defective HIV-1 vector was used. Next, we analyzed the role of ezrin in HIV-1 replication. The level of p24 protein was determined in HeLa/CD4 cells stably expressing EZ-Wt, -N, -TA, or -TD after inoculation with replication-competent HIV-1 NL4-3 (Nomaguchi et al., 2014). The level of p24 protein was significantly lower in EZ-N-expressing cells but unchanged in EZ-Wt, -TA, and -TD-expressing cells (**Figure 6A**), indicating that the EZ-N mutant inhibits HIV-1 replication. Under replication-competent conditions, p24 amount was significantly lower in siEZ-transfected cells (**Figure 6B**), suggesting that endogenous ezrin is required for the HIV-1 replication.

**FIGURE 7:**
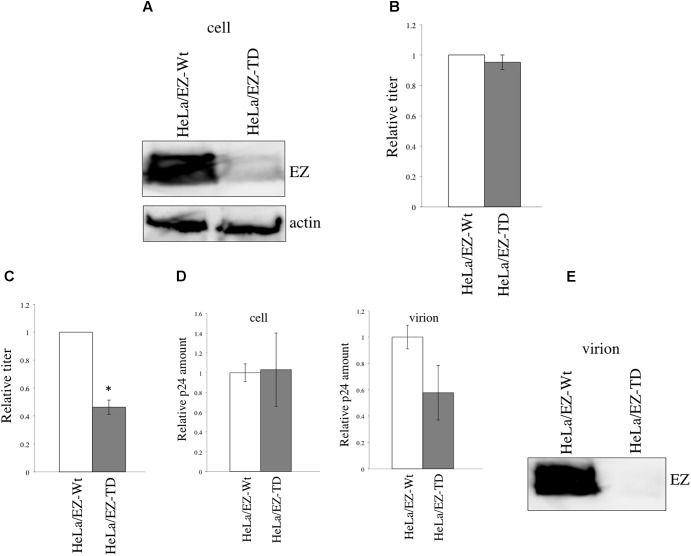
EZ-TD level is reduced during the maintenance of EZ-TD-expressing cells. **(A)** HeLa/CD4 cells stably expressing EZ-Wt or EZ-TD were cultured for long time, and cell lysates were prepared from the cells. The cell lysates were analyzed by western blotting. **(B)** Transduction titers of X4-tropic HIV-1 Env-containing HIV-1 vector were measured in the long-cultured cells. Transduction titers of the EZ-Wt-expressing cells were always set to 1, and relative values to transduction titers in the EZ-Wt-expressing cells are indicated. Error bars show standard deviations. **(C)** HeLa/CD4 cells expressing EZ-Wt or EZ-TD were transfected by the X4-tropic HIV-1 vector construction plasmids. Culture supernatants were inoculated to HeLa/CD4 cells. Transduction titers of culture supernatants from the EZ-Wt-expressing cells were always set to 1, and relative values to transduction titers from the EZ-Wt-expressing cells are indicated. Error bars show standard deviations. An asterisk indicates statistically significant difference. **(D)** The p24 levels in cell lysates or culture supernatants were measured by ELISA. Relative values to p24 amounts in the EZ-Wt-expressing cells were indicated. Error bars show standard deviations. **(E)** Virion pellets from the transfected cells were analyzed by western blotting using the VSV-G epitope antibody.

Although the above experiment indicates that the EZ-TD significantly inhibited HIV-1 virion production (**Figure 4**), EZ-TD did not affect HIV-1 replication. To understand the discrepancy, the EZ-TD protein level was again measured by western blotting. The EZ-TD level was reduced during the maintenance of EZ-TD-expressing cells (**Figure 7A**). This result supports the conclusion that EZ-TD inhibits cell growth and/or is cytotoxic. Transduction titers of HIV-1 vector in the EZ-TD-expressing cells became similar to those in the EZ-Wt-expressing cells (**Figure 7B**). When the EZ-Wt- and EZ-TD-expressing HeLa cells were transfected by HIV-1 vector construction plasmids, transduction titers of culture supernatants from the EZ-TD-expressing cells were moderately lower than those from the EZ-Wt-expressing cells (**Figure 7C**). Consistently, p24 amounts in culture supernatants from the EZ-TD-expressing cells were moderately decreased (**Figure 7D**), although p24 levels in cell lysates were similar. Because EZ-TD protein level was decreased during the culture of EZ-TD-expressing cells, HIV-1 replication was not affected in the cells.

To examine whether EZ-TD protein is incorporated into HIV-1 particles, the HeLa/CD4 cells expressing EZ-TD at a lower level were transfected by the HIV-1 vector construction plasmids, and virion pellets were prepared from the transfected cells. EZ-TD protein was not detected in virion fractions (**Figure 7E**). This result supports the conclusion that EZ-TD inhibits HIV-1 virion production.

To assess the impact of EZ-TD on HIV-1 replication, HeLa/CD4 cells were transfected by the EZ-Wt or EZ-TD expression plasmid, and were inoculated with replication-competent HIV-1 24 h after the transfection. The levels of p24 protein in culture supernatants from the EZ-TD-transfected cells were slightly lower than those from the EZ-Wt-transfected cells (**Figure 6C**). This result shows that EZ-TD has negative effect on HIV-1 replication.

## Discussion

We observed that X4-tropic HIV-1 vector infection was inhibited by expression of the EZ-TA mutant but increased by expression of the EZ-TD mutant, suggesting that ezrin phosphorylation in target cells is required for efficient HIV-1 entry. Expression of a dominant-negative mutant of ezrin (EZ-N) and ezrin silencing in HIV-1 vector-producing cells significantly reduced the infectivity of released virions without affecting virion production. This result indicates that endogenous ezrin expression is required for virion infectivity.

Previous reports have established that phosphorylation of moesin, another member of the ERM family, is important for HIV-1 infection (Barrero-Villar et al., 2009). Binding of the HIV-1 Env protein to target cells triggers clustering of CD4 and co-receptor molecules at the virion binding site on the actin cytoskeleton (Jolly et al., 2004, 2007). Accordingly, ezrin phosphorylation is thought to control the clustering of HIV-1 receptor proteins by linking the actin cytoskeleton to the receptors. However, we detected no phosphorylated ezrin in HeLa/CD4 cells inoculated with the X4-tropic HIV-1 vector. Thus, ezrin phosphorylation may occur locally and transiently after interaction between the Env and receptor proteins.

Ezrin phosphorylation is cell-line dependent. Constitutive phosphorylation of threonine 567 has been observed in Jurkat T cells, as reported (Parameswaran and Gupta, 2013). We attempted constructing Jurkat T cells stably expressing EZ-Wt, EZ-N, EZ-TA, and EZ-TD using the MLV vector, but EZ-N- and EZ-TA-expressing Jurkat cells were not obtained. This result suggests that ezrin phosphorylation is required for Jurkat cell viability. In contrast, our results indicate that ezrin is not phosphorylated in 293T, COS7, and HeLa cells, suggesting that phosphorylated ezrin may have a negative effect on these cells. Indeed, the expression level of the EZ-TD protein was lower than that of the EZ-Wt and EZ-TA proteins in HeLa cells. Although we have reported that target cell expression of ezrin is required for efficient HIV-1 infection (Kubo et al., 2008), contradictory reports show that other members of the ERM family restrict retrovirus infection (Naghavi et al., 2007; Haedicke et al., 2008; Capalbo et al., 2011; Brégnard et al., 2013; Li et al., 2016). The roles of ERM family proteins in HIV-1 infection might depend on their phosphorylation state in the cells studied.

**FIGURE 8:**
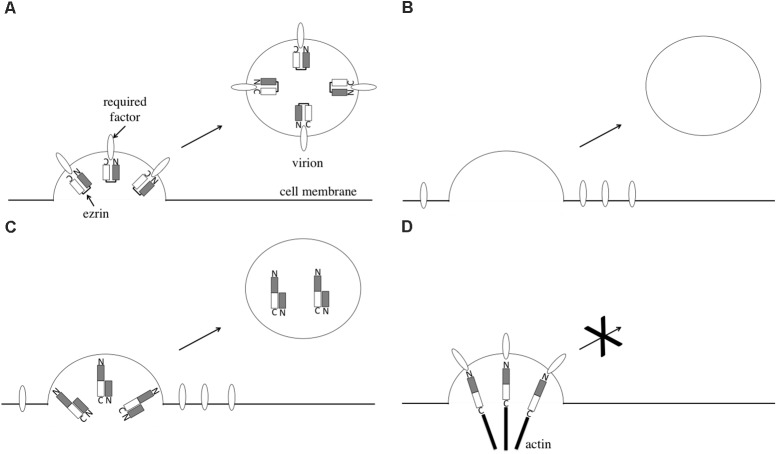
Speculated model of ezrin in HIV-1 virion production. HIV-1 virion formation in control cells **(A)**, ezrin-silenced cells **(B)**, EZ-N-expressing cells **(C)**, and EZ-TD-expressing cells **(D)** are indicated.

The incorporation of unphosphorylated ezrin into HIV-1 particles is necessary for the infectivity of the released virions. As one of the reasons, unphosphorylated ezrin may bind to a cellular factor that is required for infection by released viral particles (**Figure 8A**). We observed that HIV-1 vector particles released from ezrin-silenced cells had lower transduction titers than did those from siGFP-transfected cells, as previously reported (Roy et al., 2014). EZ-N, which functions as a dominant negative mutant of phosphorylated ezrin, was incorporated into HIV-1 particles and decreased the infectivity of the released virions. Ezrin silencing and EZ-N may inhibit incorporation of the cellular factor into virions (**Figures 8B,C**).

Phosphorylated ezrin inhibits HIV-1 virion release. EZ-TD constitutively serves as a linker between membrane proteins and the actin cytoskeleton. Thus, EZ-TD may inhibit HIV-1 virion release by tethering HIV-1 particles to the cell surface (**Figure 8D**). For example, ICAM-1 interacts with ezrin (Barreiro et al., 2002) and is incorporated into HIV-1 particles (Tardif and Tremblay, 2003). The complex consisting of ICAM-1, phosphorylated ezrin, and the actin cytoskeleton may inhibit the release of HIV-1 particles into culture supernatants. This result is consistent with our observation that phosphorylated ezrin was not detected in HIV-1 particles. If so, HIV-1 Gag protein amount would increase in the EZ-TD-expressing cells. However, the viral protein levels were not altered by EZ-TD. Tetherin also inhibits HIV-1 particle production, but Gag protein levels are similar in the presence and absence of tetherin (Neil et al., 2008). The Gag protein may be degraded in lysosome, as already reported (Miyakawa et al., 2009).

We observed here that EZ-N expression and ezrin silencing significantly inhibit HIV-1 replication and, as we reported previously, attenuate HIV-1 entry. In addition, EZ-N expression and ezrin silencing in HIV-1-producing cells decrease the infectivity of released HIV-1 particles. Thus, EZ-N expression and ezrin silencing significantly inhibit HIV-1 replication. Expression of the EZ-TA mutant in target cells moderately inhibited X4-tropic HIV-1 Env-mediated infection but not HIV-1 replication. The negative effect of EZ-TA on HIV-1 infection is not enough to inhibit HIV-1 replication. The expression of EZ-TD in target cells increased HIV-1 infection but inhibited HIV-1 virion production. EZ-TD expression moderately inhibited HIV-1 replication. The negative effect of EZ-TD on HIV-1 virion production is predominant to the positive effect on HIV-1 entry.

Taken together, our results indicate that ezrin is involved in many steps of the HIV-1 life cycle, including entry, virion production, and infectivity of the released particles. Further study is needed to determine the molecular mechanisms underlying these observations.

## Author Contributions

HK, MI, YU, and YK performed the experiments in this study. HK, HH, TM, and YK analyzed the data. YK wrote the manuscript.

## Conflict of Interest Statement

The authors declare that the research was conducted in the absence of any commercial or financial relationships that could be construed as a potential conflict of interest.

## Supplementary Material

FIGURE S1 Cellular localization of HIV-1 Gag and ezrin proteins. COS7 cells were transfected with the HIV-1 vector construction plasmids together with the EZ-Wt or EZ-TD expression plasmid, and permeabilized with methanol. The cells were first stained with mouse anti-VSV-G epitope and rabbit anti-HIV-1 p24 antibodies, and then with Cy3-conjugated anti-mouse IgG and FITC-conjugated anti-rabbit IgG antibodies. The cells were observed under a laser confocal microscope. Representative results are shown.
