# Supplementary figures and images for: Role of Ezrin Phosphorylation in HIV-1 Replication
Source: Front Microbiol. 2018 Aug 27;9:1912. doi: 10.3389/fmicb.2018.01912 (PMC6119696; doi:10.3389/fmicb.2018.01912)

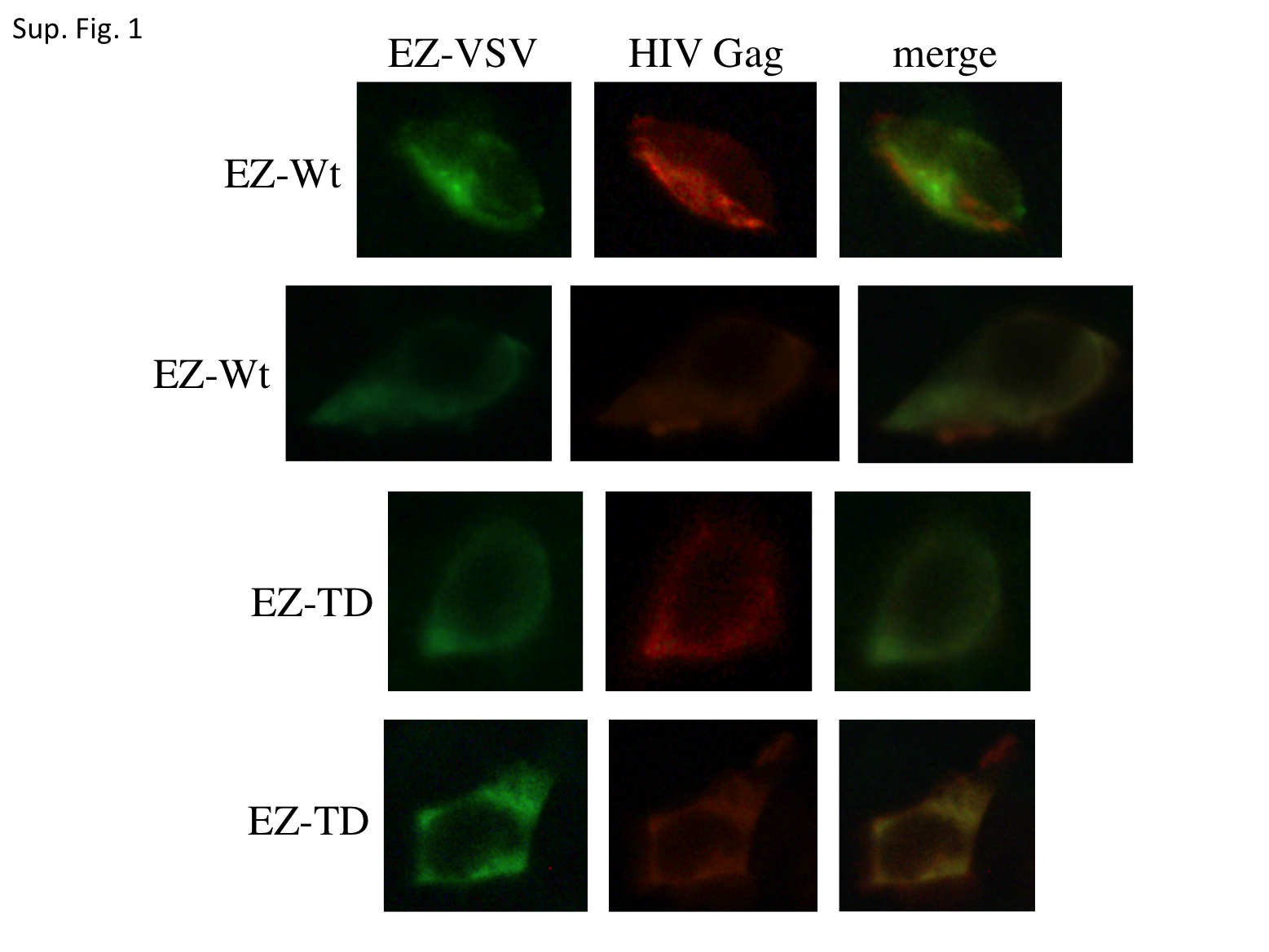

Supplement: FIGURE S1 — Cellular localization of HIV-1 Gag and ezrin proteins. COS7 cells were transfected with the HIV-1 vector construction plasmids together with the EZ-Wt or EZ-TD expression plasmid, and permeabilized with methanol. The cells were first stained with mouse anti-VSV-G epitope and rabbit anti-HIV-1 p24 antibodies, and then with Cy3-conjugated anti-mouse IgG and FITC-conjugated anti-rabbit IgG antibodies. The cells were observed under a laser confocal microscope. Representative results are shown. [file Image_1.TIF]
